# Supplementary material for: Comparison of three variant callers for human whole genome sequencing
Source: Sci Rep. 2018 Dec 14;8:17851. doi: 10.1038/s41598-018-36177-7 (PMC6294778; doi:10.1038/s41598-018-36177-7)

Comparison of three variant callers for human whole genome sequencing

Supplementary informatin

Anna Supernat[1], Oskar Valdimar Vidarsson[2], Vidar M. Steen[3, 4], Tomasz Stokowy[2, 3, 4,*]

[1]Laboratory of Cell Biology, Intercollegiate Faculty of Biotechnology, University of Gdańsk and Medical University of Gdańsk, Poland

[2]Computational Biology Unit, Institute of Informatics, University of Bergen, Norway

[3]NORMENT & K.J. Jebsen Centre for Psychosis Research, Department of Clinical Science, University of Bergen, Norway

[4]Dr. E. Martens Research Group for Biological Psychiatry, Department of Medical Genetics, Haukeland University Hospital, Bergen, Norway

* To whom correspondence should be addressed.
Tel: +47 48 61 42 19
Email: tomasz.stokowy@k2.uib.no

Index

Page 2	Supplementary Information 1
Page 18	Supplementary Information 2
Page 34	Supplementary Information 3
Page 35	Supplementary Information 4
Page 36	Supplementary Information 5

Supplementary Information 1 – Literature Search

To obtain a comprehensive overview of applications of WGS in clinical settings, we performed a non-systematic literature search (NCBI PubMed) on 29/03/2018 using the following terms: [“clinical use” OR “clinical application” OR “genetic testing” OR “clinical genetics”] AND [“human whole genome sequencing OR “human WGS”]. The search returned 3877 PubMed records. We further restricted the output to "Journal Articles" that appeared during the last year (i.e. after 30/03/2017), with additional filtering for species category: humans. This selection procedure returned 512 records (listed below).
Out of the 512 returned papers, 37 turned out to be reviews, 110 were Whole Exome Sequencing-based, 114 included studies related to diagnosis of infectious diseases and 140 included articles focusing on bioinformatics tools description, method validation, quality assessment, surveys or animal studies. The remaining articles contained original WGS-based data on causal genetic variants in hereditary diseases or cancer (N=50 and N=35, respectively). Furthermore, six papers referred to prenatal, newborn or preimplantation screening, while five papers covered population studies, pharmacogenetics or forensic medicine, and two were related to the CRISPR system.

Listed below are the 512 relevant records of the literature search:

1. DNA extraction from primary liquid blood cultures for bloodstream infection diagnosis using whole genome sequencing. J Med Microbiol. 2018
2. [Current situation and prospect of breast cancer liquid biopsy]. Zhonghua Wai Ke Za Zhi. 2018
3. Discordant congenital Zika syndrome twins show differential in vitro viral susceptibility of neural progenitor cells. Nat Commun. 2018
4. Identification of co-occurrence in a patient with Dent's disease andADA2-deficiency by exome sequencing. Gene. 2018
5. Whole exome sequencing in neurogenetic odysseys: An effective, cost- and time-saving diagnostic approach. PLoS One. 2018
6. Comparative genomics reveals differences in mobile virulence genes of Escherichia coli O103 pathotypes of bovine fecal origin. PLoS One. 2018
7. [From a Ph.D. Thesis: Understanding the Past, Predicting the Future]. Yakugaku Zasshi. 2018
8. Whole genome sequencing analysis for cancer genomics and precision medicine. Cancer Sci. 2018
9. Elucidating the genomic architecture of Asian EGFR-mutant lung adenocarcinoma through multi-region exome sequencing. Nat Commun. 2018
10. Comprehensive molecular profiling of advanced/metastatic olfactory neuroblastomas. PLoS One. 2018
11. Integrative genomic and transcriptomic analysis of leiomyosarcoma. Nat Commun. 2018
12. Punctuated evolution of canonical genomic aberrations in uveal melanoma. Nat Commun. 2018
13. Diagnostic outcomes of exome sequencing in patients with syndromic or non-syndromic hearing loss. PLoS One. 2018
14. SLC52A2 mutations cause SCABD2 phenotype: A second report. Int J Pediatr Otorhinolaryngol. 2018
15. Identification of two novel pathogenic compound heterozygous MYO7A mutations in Usher syndrome by whole exome sequencing. Int J Pediatr Otorhinolaryngol. 2018
16. TSC1 Mutations in Keratoconus Patients With or Without Tuberous Sclerosis. Invest Ophthalmol Vis Sci. 2017
17. Sequence data and association statistics from 12,940 type 2 diabetes cases and controls. Sci Data. 2017
18. Streptococcal toxic shock syndrome caused by the dissemination of an invasive emm3/ST15 strain of Streptococcus pyogenes. BMC Infect Dis. 2017
19. Whole exome sequencing to identify genetic markers for trastuzumab-induced cardiotoxicity. Cancer Sci. 2018
20. CELSR2 is a candidate susceptibility gene in idiopathic scoliosis. PLoS One. 2017
21. Whole-genome analyses of human adenovirus type 55 emerged in Tibet, Sichuan and Yunnan in China, in 2016. PLoS One. 2017
22. Route of infection alters virulence of neonatal septicemia Escherichia coli clinical isolates. PLoS One. 2017
23. De Novo Variants in GRIA4 Lead to Intellectual Disability with or without Seizures and Gait Abnormalities.

Am J Hum Genet. 2017
24. Deep sequencing of near full-length HIV-1 genomes from plasma identifies circulating subtype C and infrequent occurrence of AC recombinant form in Southern India. PLoS One. 2017
25. Molecular characterization of hepatitis B virus in Bangladesh reveals a highly recombinant population. PLoS One. 2017
26. Breast cancer: The translation of big genomic data to cancer precision medicine. Cancer Sci. 2018
27. Homozygous GRID2 missense mutation predicts a shift in the D-serine binding domain of GluD2 in a case with generalized brain atrophy and unusual clinical features. BMC Med Genet. 2017
28. A New Zealand platform to enable genetic investigation of adverse drug reactions. N Z Med J. 2017
29. The exomic landscape of t(14;18)-negative diffuse follicular lymphoma with 1p36 deletion. Br J Haematol. 2018
30. RYR1 causing distal myopathy. Mol Genet Genomic Med. 2017
31. Panel-based whole exome sequencing identifies novel mutations in microphthalmia and anophthalmia patients showing complex Mendelian inheritance patterns. Mol Genet Genomic Med. 2017
32. The genetic profile of Leber congenital amaurosis in an Australian cohort. Mol Genet Genomic Med. 2017
33. A novel splice site variant in CYP11A1 in trans with the p.E314K variant in a male patient with congenital adrenal insufficiency. Mol Genet Genomic Med. 2017
34. Shiga Toxin-Producing E. coli Infections Associated with Flour. N Engl J Med. 2017
35. Elucidating the pathogenesis of synchronous and metachronous tumors in a woman with endometrioid carcinomas using a whole-exome sequencing approach. Cold Spring Harb Mol Case Stud. 2017
36. Novel genes associated with amyotrophic lateral sclerosis: diagnostic and clinical implications. Lancet Neurol. 2018
37. Mutations in RAB39B in individuals with intellectual disability, autism spectrum disorder, and macrocephaly. Mol Autism. 2017
38. Occurrence of Enterobacter hormaechei carrying blaNDM-1 and blaKPC-2 in China. Diagn Microbiol Infect Dis. 2018
39. First case report of Cohen syndrome in the Tunisian population caused by VPS13B Mutations. BMC Med Genet. 2017
40. Molecular and epidemiological characterization of carbapenemase-producing Enterobacteriaceae in Norway, 2007 to 2014. PLoS One. 2017
41. COPA syndrome in an Icelandic family caused by a recurrent missense mutation in COPA. BMC Med Genet. 2017
42. Clinical delineation of a subtype of frontonasal dysplasia with creased nasal ridge and upper limb anomalies: Report of six unrelated patients. Am J Med Genet A. 2017
43. Retention of Interstitial Genes between TMPRSS2 and ERG Is Associated with Low-Risk Prostate Cancer. Cancer Res. 2017
44. Rapid and Accurate Sequencing of Enterovirus Genomes Using MinION Nanopore Sequencer. Biomed Environ Sci. 2017
45. Efficacy and safety of trametinib in Japanese patients with advanced biliary tract cancers refractory to gemcitabine. Cancer Sci. 2018
46. Spatio-Temporal Genomic Heterogeneity, Phylogeny, and Metastatic Evolution in Salivary Adenoid Cystic Carcinoma. J Natl Cancer Inst. 2017.
47. Genomic sequencing identifies a few mutations driving the independent origin of primary liver tumors in a chronic hepatitis murine model. PLoS One. 2017
48. Whole exome sequencing in thrombophilic pedigrees to identify genetic risk factors for venous thromboembolism. PLoS One. 2017
49. Time for change: a new training programme for morpho-molecular pathologists? J Clin Pathol. 2018
50. Impact of extensive antibiotic treatment on faecal carriage of antibiotic-resistant enterobacteria in children in a low resistance prevalence setting. PLoS One.
51.Drivers of Tuberculosis Transmission. J Infect Dis. 2017
52. Mutations in GPAA1, Encoding a GPI Transamidase Complex Protein, Cause Developmental Delay, Epilepsy, Cerebellar Atrophy, and Osteopenia. Am J Hum Genet. 2017
53. De Novo Mutations in Protein Kinase Genes CAMK2A and CAMK2B Cause Intellectual Disability. Am J Hum Genet. 2017
54. High Rate of Recurrent De Novo Mutations in Developmental and Epileptic Encephalopathies. Am J Hum Genet. 2017
55. A novel FLNC frameshift and an OBSCN variant in a family with distal muscular dystrophy. PLoS One. 2017
56. Preterm Infant-Associated Clostridium tertium, Clostridium cadaveris, and Clostridium paraputrificum Strains: Genomic and Evolutionary Insights. Genome Biol Evol. 2017

57. A comparison of extended spectrum β-lactamase producing Escherichia coli from clinical, recreational water and wastewater samples associated in time and location. PLoS One. 2017
58. Identification and analysis of the genetic causes in nine unrelated probands with syndromic craniosynostosis. Gene. 2018
59. Prevalence and clonality of synchronous primary carcinomas in the bladder and prostate. J Pathol. 2018
60. Whole genome sequencing for the molecular characterization of carbapenem-resistant Klebsiella pneumoniae strains isolated at the Italian ASST Fatebenefratelli Sacco Hospital, 2012-2014. BMC Infect Dis. 2017
61. Genetic diversity and worldwide distribution of the deltavirus genus: A study of 2,152 clinical strains. Hepatology. 2017
62. Homozygous indel mutation in CDH11 as the probable cause of Elsahy-Waters syndrome. Am J Med Genet A. 2017
63. Genetic and Functional Drivers of Diffuse Large B Cell Lymphoma. Cell. 2017
64. Genomic Organization of TBK1 Copy Number Variations in Glaucoma Patients. J Glaucoma. 2017
65. Mycobacterium grossiae sp. nov., a rapidly growing, scotochromogenic species isolated from human clinical respiratory and blood culture specimens. Int J Syst Evol Microbiol. 2017
66. STRScan: targeted profiling of short tandem repeats in whole-genome sequencing data. BMC Bioinformatics. 2017
67. [Mutational analysis and prenatal diagnosis in a family affected with hypophosphatemic rickets]. Zhonghua Yi Xue Yi Chuan Xue Za Zhi. 2017
68. Full-length mutation search of the TP53 gene in acute myeloid leukemia has increased significance as a prognostic factor. Ann Hematol. 2018
69. Exome-wide association study reveals novel psoriasis susceptibility locus at TNFSF15 and rare protective alleles in genes contributing to type I IFN signalling. Hum Mol Genet. 2017
70. Identification of a novel synaptic protein, TMTC3, involved in periventricular nodular heterotopia with intellectual disability and epilepsy. Hum Mol Genet. 2017
71. Use of Exome Sequencing for Infants in Intensive Care Units: Ascertainment of Severe Single-Gene Disorders and Effect on Medical Management. JAMA Pediatr. 2017
72. Clinical significance of BRAF non-V600E mutations on the therapeutic effects of anti-EGFR monoclonal antibody treatment in patients with pretreated metastatic colorectal cancer: the Biomarker Research for anti-EGFR monoclonal Antibodies by Comprehensive Cancer genomics (BREAC) study. Br J Cancer. 2017
73. Clonality, Heterogeneity, and Evolution of Synchronous Bilateral Ovarian Cancer. Cancer Res. 2017
74. Phenotypic Characterization of EIF2AK4 Mutation Carriers in a Large Cohort of Patients Diagnosed Clinically With Pulmonary Arterial Hypertension. Circulation. 2017
75. Evaluating the Impact of Functional Genetic Variation on HIV-1 Control. J Infect Dis. 2017
76. The genomic and epigenomic landscape in thymic carcinoma. Carcinogenesis. 2017
77. Exploring genome-wide DNA methylation patterns in Aicardi syndrome. Epigenomics. 2017
78. Hypomorphic Recessive Variants in SUFU Impair the Sonic Hedgehog Pathway and Cause Joubert Syndrome with Cranio-facial and Skeletal Defects. Am J Hum Genet. 2017
79. Novel NR2F1 variants likely disrupt DNA binding: molecular modeling in two cases, review of published cases, genotype-phenotype correlation, and phenotypic expansion of the Bosch-Boonstra-Schaaf optic atrophy syndrome. Cold Spring Harb Mol Case Stud. 2017
80. Laboratory-acquired infections of Salmonella enterica serotype Typhi in South Africa: phenotypic and genotypic analysis of isolates.BMC Infect Dis. 2017
81. Exome Sequencing Provides Evidence of Polygenic Adaptation to a Fat-Rich Animal Diet in Indigenous Siberian Populations. Mol Biol Evol. 2017
82. Genetic heterogeneity of uncharacterized childhood autoimmune diseases with lymphoproliferation.Pediatr Blood Cancer. 2018
83. Epidemiological characterization of a nosocomial outbreak of extended spectrum β-lactamase Escherichia coli ST-131 confirms the clinical value of core genome multilocus sequence typing. APMIS. 2017
84. Super-Enhancers Promote Transcriptional Dysregulation in Nasopharyngeal Carcinoma.Cancer Res. 2017
85. Genomic signatures as predictive biomarkers of homologous recombination deficiency in ovarian cancer. Eur J Cancer. 2017
86. Identification of the genetic determinants responsible for retinal degeneration in families of Mexican descent. Ophthalmic Genet. 2018
87. Loss-of-function variants in NFIA provide further support that NFIA is a critical gene in 1p32-p31 deletion syndrome: A four patient series. Am J Med Genet A. 2017
88. Leptomonas seymouri narna-like virus 1 and not leishmaniaviruses detected in kala-azar samples from India. Arch Virol. 2017
89. Rapid Targeted Genomics in Critically Ill Newborns. Pediatrics. 2017

90. SHORT syndrome due to a novel de novo mutation in PRKCE (Protein Kinase Cε) impairing TORC2-dependent AKT activation. Hum Mol Genet. 2017
91. Whole genome characterization of sequence diversity of 15,220 Icelanders. Sci Data. 2017
92. Molecular Evolution of Herpes Simplex Virus 2 Complete Genomes: Comparison between Primary and Recurrent Infections. J Virol. 2017
93. Early genetic aberrations in patients with sporadic colorectal cancer. Mol Carcinog. 2018
94. Relatedness of wildlife and livestock avian isolates of the nosocomial pathogen Acinetobacter baumannii to lineages spread in hospitals worldwide. Environ Microbiol. 2017
95. Real-time genomic investigation underlying the public health response to a Shiga toxin-producing Escherichia coli O26:H11 outbreak in a nursery. Epidemiol Infect. 2017
96. Germline MSH6 Mutation in a Patient With Two Independent Primary Glioblastomas. J Neuropathol Exp Neurol. 2017
97. Controversy and debate on clinical genomics sequencing-paper 2: clinical genome-wide sequencing: don't throw out the baby with the bathwater! J Clin Epidemiol. 2017
98. Mutations of conserved non-coding elements of PITX2 in patients with ocular dysgenesis and developmental glaucoma. Hum Mol Genet. 2017
99. Identification of causative variants in TXNL4A in Burn-McKeown syndrome and isolated choanal atresia. Eur J Hum Genet. 2017
100. Whole-Genome Sequencing Reveals Breast Cancers with Mismatch Repair Deficiency. Cancer Res. 2017
101. The pathogenicity of genomic/genetic variant of X-chromosomal genes in males with intellectual disability. Yi Chuan. 2017
102. Modest heterologous protection after Plasmodium falciparum sporozoite immunization: a double-blind randomized controlled clinical trial. BMC Med. 2017
103. [Advances in genetic research of cerebral palsy]. Zhongguo Dang Dai Er Ke Za Zhi. 2017
104. Emerging peak on the phylogeographic landscape of Mycobacterium tuberculosis in West Asia: Definitely smoke, likely fire. Mol Phylogenet Evol. 2017
105. Exome Sequencing Landscape Analysis in Ovarian Clear Cell Carcinoma Shed Light on Key Chromosomal Regions and Mutation Gene Networks. Am J Pathol. 2017
106. HPV16 E7 Genetic Conservation Is Critical to Carcinogenesis. Cell. 2017
107. SEXCMD: Development and validation of sex marker sequences for whole-exome/genome and RNA sequencing. PLoS One. 2017
108. Validation of Next-Generation Sequencer for 24-Chromosome Aneuploidy Screening in Human Embryos. Genet Test Mol Biomarkers. 2017
109. Efficient detection of chromosome imbalances and single nucleotide variants using targeted sequencing in the clinical setting. Eur J Med Genet. 2017
110. Whole genome sequencing of ESBL-producing Escherichia coli isolated from patients, farm waste and canals in Thailand. Genome Med. 2017
111. Unraveling genetic predisposition to familial or early onset gastric cancer using germline whole-exome sequencing. Eur J Hum Genet. 2017
112. Clinical practice recommendations for treatment with active vitamin D analogues in children with chronic kidney disease Stages 2-5 and on dialysis. Nephrol Dial Transplant. 2017
113. Clinical practice recommendations for native vitamin D therapy in children with chronic kidney disease Stages 2-5 and on dialysis. Nephrol Dial Transplant. 2017
114. Controversy and debate on clinical genomics sequencing-paper 1: genomics is not exceptional: rigorous evaluations are necessary for clinical applications of genomic sequencing. J Clin Epidemiol. 2017
115. Exome sequencing for the differential diagnosis of ciliary chondrodysplasias: Example of a WDR35 mutation case and review of the literature. Eur J Med Genet. 2017
116. Establishing the role of rare coding variants in known Parkinson's disease risk loci. Neurobiol Aging. 2017
117. Maternal trans-general analysis of the human mitochondrial DNA pattern. Biochem Biophys Res Commun. 2017
118. Assessing the accuracy of blood RNA profiles to identify patients with post-concussion syndrome: A pilot study in a military patient population. PLoS One. 2017
119. Improving power of association tests using multiple sets of imputed genotypes from distributed reference panels.Genet Epidemiol. 2017
120. Molecular characterization of hepatitis B virus in Vietnam. BMC Infect Dis. 2017
121. GABBR2 mutations determine phenotype in rett syndrome and epileptic encephalopathy. Ann Neurol. 2017
122. Clinical implications of neoepitope landscapes for adult and pediatric cancers. Genome Med. 2017
123. Segregation of a novel p.(Ser270Tyr) MAF mutation and p.(Tyr56*) CRYGD variant in a family with dominantly inherited congenital cataracts. Mol Biol Rep. 2017

124. Neutropenia in Patients with Common Variable Immunodeficiency: a Rare Event Associated with Severe Outcome. J Clin Immunol. 2017
125. Chronological age prediction based on DNA methylation: Massive parallel sequencing and random forest regression. Forensic Sci Int Genet. 2017
126. Key signaling pathways in thyroid cancer. J Endocrinol. 2017
127. Epigenome-Wide Association Study Identifies Cardiac Gene Patterning and a Novel Class of Biomarkers for Heart Failure. Circulation. 2017
128. Prolonged Shedding of Human Coronavirus in Hematopoietic Cell Transplant Recipients: Risk Factors and Viral Genome Evolution.J Infect Dis. 2017
129. SweGen: a whole-genome data resource of genetic variability in a cross-section of the Swedish population. Eur J Hum Genet. 2017
130. Rational confederation of genes and diseases: NGS interpretation via GeneCards, MalaCards and VarElect. Biomed Eng Online. 2017
131. Genetic diagnosis of polycystic kidney disease, Alport syndrome, and thalassemia minor in a large Chinese family. Clin Sci (Lond). 2017
132. Advances in basic and clinical immunology in 2016. J Allergy Clin Immunol. 2017
133. A Supervised Statistical Learning Approach for Accurate Legionella pneumophila Source Attribution during Outbreaks. Appl Environ Microbiol. 2017
134. Retinopathy and optic atrophy: Expanding the phenotypic spectrum of pathogenic variants in the AARS2 gene. Ophthalmic Genet. 2018
135. Stakeholders in psychiatry and their attitudes toward receiving pertinent and incident findings in genomic research. Am J Med Genet A. 2017
136. Cystic kidneys in fetal Walker-Warburg syndrome with POMT2 mutation: Intrafamilial phenotypic variability in four siblings and review of literature. Am J Med Genet A. 2017
137. Autosomal recessive cone-rod dystrophy can be caused by mutations in the ATF6 gene. Eur J Hum Genet. 2017
138. Leveraging network analytics to infer patient syndrome and identify causal genes in rare disease cases. BMC Genomics. 2017
139. Reducing diagnostic turnaround times of exome sequencing for families requiring timely diagnoses. Eur J Med Genet. 2017
140. Two Cases of Multi-antibiotic Resistant Cronobacter spp. Infections of Infants in China. Biomed Environ Sci. 2017
141. Lack of FOXE3 coding mutation in a case of congenital aphakia.Ophthalmic Genet. 2018
142. Settling the score: variant prioritization and Mendelian disease. Nat Rev Genet. 2017
143. Utility of rapid whole-exome sequencing in the diagnosis of Niemann-Pick disease type C presenting with fetal hydrops and acute liver failure. Cold Spring Harb Mol Case Stud. 2017
144. Next generation sequencing reveals the antibiotic resistant variants in the genome of Pseudomonas aeruginosa. PLoS One. 2017
145. Exome sequencing in schizophrenic patients with high levels of homozygosity identifies novel and extremely rare mutations in the GABA/glutamatergic pathways. PLoS One. 2017
146. BLOOM: BLoom filter based oblivious outsourced matchings. BMC Med Genomics. 2017
147. Integrative clinical genomics of metastatic cancer. Nature. 2017
148. Retrospective use of next-generation sequencing reveals the presence of Enteroviruses in acute influenza-like illness respiratory samples collected in South/South-East Asia during 2010-2013. J Clin Virol. 2017
149. Experimental Modeling Supports a Role for MyBP-HL as a Novel Myofilament Component in Arrhythmia and Dilated Cardiomyopathy. Circulation. 2017
150. A novel mutation in CDK5RAP2 gene causes primary microcephaly with speech impairment and sparse eyebrows in a consanguineous Pakistani family. Eur J Med Genet. 2017
151. The invasome of Salmonella Dublin as revealed by whole genome sequencing. BMC Infect Dis. 2017
152. Whole-Exome Sequencing Identifies the 6q12-q16 Linkage Region and a Candidate Gene, TTK, for Pulmonary Nontuberculous Mycobacterial Disease. Am J Respir Crit Care Med. 2017
153. Identification of Novel Breast Cancer Risk Loci. Cancer Res. 2017
154. Large intragenic deletion of CDC73 (exons 4-10) in a three-generation hyperparathyroidism-jaw tumor (HPT-JT) syndrome family.BMC Med Genet. 2017
155. Mechanisms by which Porphyromonas gingivalis evades innate immunity. PLoS One. 2017
156. Contribution of exome sequencing for genetic diagnostic in arrhythmogenic right ventricular cardiomyopathy/dysplasia. PLoS One. 2017
157. Low-Frequency Synonymous Coding Variation in CYP2R1 Has Large Effects on Vitamin D Levels and Risk of Multiple Sclerosis. Am J Hum Genet. 2017
158. Association Analysis of the MHC in Lupus Nephritis. J Am Soc Nephrol. 2017

159. Rare Genome-Wide Copy Number Variation and Expression of Schizophrenia in 22q11.2 Deletion Syndrome. Am J Psychiatry. 2017.
160. Application of whole-exome sequencing to direct the specific functional testing and diagnosis of rare inherited bleeding disorders in patients from the Öresund Region, Scandinavia. Br J Haematol. 2017
161. The prevalence of DICER1 pathogenic variation in population databases. Int J Cancer. 2017
162. Targeted Genome Sequencing Reveals Varicella-Zoster Virus Open Reading Frame 12 Deletion. J Virol. 2017
163. Regions of common inter-individual DNA methylation differences in human monocytes: genetic basis and potential function. Epigenetics Chromatin. 2017
164. Whole exome sequencing unveils a frameshift mutation in CNGB3 for cone dystrophy: A case report of an Indian family. Medicine (Baltimore). 2017
165. Limitations of the Mycobacterium tuberculosis reference genome H37Rv in the detection of virulence-related loci. Genomics. 2017
166. Evidence for genetic association between chromosome 1q loci and predisposition to colorectal neoplasia. Br J Cancer. 2017
167. Differentiating Botulinum Neurotoxin-Producing Clostridia with a Simple, Multiplex PCR Assay. Appl Environ Microbiol. 2017
168. Exiguobacterium sp. A1b/GX59 isolated from a patient with community-acquired pneumonia and bacteremia: genomic characterization and literature review. BMC Infect Dis. 2017
169. Drug discovery and development for rare genetic disorders. Am J Med Genet A. 2017
170. Baby genome screening: paving the way to genetic discrimination? BMJ. 2017
171. Genomic and transcriptomic heterogeneity of colorectal tumours arising in Lynch syndrome. J Pathol. 2017
172. The whole-genome landscape of medulloblastoma subtypes.Nature. 2017 Jul
173. Whole genome sequencing identifies a novel ALMS1 gene mutation in two Chinese siblings with Alström syndrome. BMC Med Genet. 2017
174. Molecular Epidemiology of Staphylococcus aureus Skin and Soft Tissue Infections in the Lao People's Democratic Republic. Am J Trop Med Hyg. 2017
175. Clinical study of genomic drivers in pancreatic ductal adenocarcinoma. Br J Cancer. 2017
176. Mutations in TYROBP are not a common cause of dementia in a Turkish cohort. Neurobiol Aging. 2017
177. Rapid identification of a Mycobacterium tuberculosis full genetic drug resistance profile through whole genome sequencing directly from sputum. Int J Infect Dis. 2017
178. Rare coding variants in PLCG2, ABI3, and TREM2 implicate microglial-mediated innate immunity in Alzheimer's disease. Nat Genet. 2017
179. Genetic and epigenetic drivers of neuroendocrine tumours (NET). Endocr Relat Cancer. 2017
180. Screening for TMEM230 mutations in young-onset Parkinson's disease. Neurobiol Aging. 2017
181. BCR-ABL1-like acute lymphoblastic leukaemia: From bench to bedside. Eur J Cancer. 2017
182. The Epithelial Sodium Channel Is a Modifier of the Long-Term Nonprogressive Phenotype Associated with F508del CFTR Mutations. Am J Respir Cell Mol Biol. 2017
183. Isolation and Whole-genome Sequence Analysis of the Imipenem Heteroresistant Acinetobacter baumannii Clinical Isolate HRAB-85. Int J Infect Dis. 2017
184. Molecular investigation by whole exome sequencing revealed a high proportion of pathogenic variants among Thai victims of sudden unexpected death syndrome. PLoS One. 2017
185. Genomic analysis of an infant with intractable diarrhea and dilated cardiomyopathy. Cold Spring Harb Mol Case Stud. 2017
186. Copy-Number Variants Detection by Low-Pass Whole-Genome Sequencing. Curr Protoc Hum Genet. 2017
187. Germline Loss-of-Function Mutations in EPHB4 Cause a Second Form of Capillary Malformation-Arteriovenous Malformation (CM-AVM2) Deregulating RAS-MAPK Signaling. Circulation. 2017
188. Rare variants of small effect size in neuronal excitability genes influence clinical outcome in Japanese cases of SCN1A truncation-positive Dravet syndrome. PLoS One. 2017
189. Interpreting whole genome and exome sequencing data of individual gastric cancer samples. BMC Genomics. 2017
190. DNA isolation protocol effects on nuclear DNA analysis by microarrays, droplet digital PCR, and whole genome sequencing, and on mitochondrial DNA copy number estimation. PLoS One. 2017
191. Association of NOD2 Mutations with Aggressive Periodontitis. J Dent Res. 2017
192. Evolution of the Staphylococcus argenteus ST2250 Clone in Northeastern Thailand Is Linked with the Acquisition of Livestock-Associated Staphylococcal Genes. MBio. 2017
193. An unusual genomic variant of pancreatic ductal adenocarcinoma with an indolent clinical course. Cold Spring Harb Mol Case Stud. 2017

194. Metastatic triple-negative breast cancer patient with TP53 tumor mutation experienced 11 months progression-free survival on bortezomib monotherapy without adverse events after ending standard treatments with grade 3 adverse events. Cold Spring Harb Mol Case Stud. 2017
195. Dissemination of Carbapenem-resistant Klebsiella pneumoniae clinical isolates with various combinations of Carbapenemases (KPC-2, NDM-1, NDM-4, and OXA-48) and 16S rRNA Methylases (RmtB and RmtC) in Vietnam. BMC Infect Dis. 2017
196. Clinical genome sequencing and population preferences for information about 'incidental' findings-From medically actionable genes (MAGs) to patient actionable genes (PAGs). PLoS One. 2017
197. SEQSpark: A Complete Analysis Tool for Large-Scale Rare Variant Association Studies Using Whole-Genome and Exome Sequence Data. Am J Hum Genet. 2017
198. Driving to Cancer on a Four-Lane Expressway. Trends Genet. 2017
199. Whole-exome sequencing identifies an α-globin cluster triplication resulting in increased clinical severity of β-thalassemia. Cold Spring Harb Mol Case Stud. 2017
200. Genomic Epidemiology of a Major Mycobacterium tuberculosis Outbreak: Retrospective Cohort Study in a Low-Incidence Setting Using Sparse Time-Series Sampling. J Infect Dis. 2017
201. Genomic investigation of a suspected outbreak of Legionella pneumophila ST82 reveals undetected heterogeneity by the present gold-standard methods, Denmark, July to November 2014. Euro Surveill. 2017
202. Landscape and variation of novel retroduplications in 26 human populations. PLoS Comput Biol. 2017
203. Multistate Outbreak of Salmonella Anatum Infections Linked to Imported Hot Peppers - United States, May-July 2016. MMWR Morb Mortal Wkly Rep. 2017
204. SLMSuite: a suite of algorithms for segmenting genomic profiles. BMC Bioinformatics. 2017.
205. A Novel Homozygous SACS Mutation Identified by Whole-Exome Sequencing in a Consanguineous Family with Autosomal Recessive Spastic Ataxia of Charlevoix-Saguenay. Cytogenet Genome Res. 2017
206. The past decade in bench research into pulmonary infectious diseases: What do clinicians need to know? Respirology. 2017
207. Systematic biobanking, novel imaging techniques, and advanced molecular analysis for precise tumor diagnosis and therapy: The Polish MOBIT project. Adv Med Sci. 2017
208. Genotype-phenotype evaluation of MED13L defects in the light of a novel truncating and a recurrent missense mutation. Eur J Med Genet. 2017
209. Whole-genome sequencing of monozygotic twins discordant for schizophrenia indicates multiple genetic risk factors for schizophrenia. J Genet Genomics. 2017
210. Decreased Fitness and Virulence in ST10 Escherichia coli Harboring blaNDM-5 and mcr-1 against a ST4981 Strain with blaNDM-5. Front Cell Infect Microbiol. 2017
211. First environmental sample containing plasmid-mediated colistin-resistant ESBL-producing Escherichia coli detected in Norway. APMIS. 2017
212. Same-day genomic and epigenomic diagnosis of brain tumors using real-time nanopore sequencing. Acta Neuropathol. 2017
213. Clonal Hematopoiesis and Risk of Atherosclerotic Cardiovascular Disease. N Engl J Med. 2017
214. Genome-wide linkage and haplotype sharing analysis implicates the MCDR3 locus as a candidate region for a developmental macular disorder in association with digit abnormalities. Ophthalmic Genet. 2017
215. Working toward precision medicine: Predicting phenotypes from exomes in the Critical Assessment of Genome Interpretation (CAGI) challenges. Hum Mutat. 2017
216. ALPK3 gene mutation in a patient with congenital cardiomyopathy and dysmorphic features. Cold Spring Harb Mol Case Stud. 2017
217. Outbreak of non-tuberculous mycobacteria skin or soft tissue infections associated with handling fish - New York City, 2013-2014. Epidemiol Infect. 2017
218. Clonal Clusters and Virulence Factors of Group C and G Streptococcus Causing Severe Infections, Manitoba, Canada, 2012-2014. Emerg Infect Dis. 2017
219. Evaluation of whole exome sequencing by targeted gene sequencing and Sanger sequencing. Clin Chim Acta. 2017
220. Whole-genome sequencing for identification of the source in hospital-acquired Legionnaires' disease. J Hosp Infect. 2017
221. Minimal genetic change in Vibrio cholerae in Mozambique over time: Multilocus variable number tandem repeat analysis and whole genome sequencing. PLoS Negl Trop Dis. 2017
222. Further delineation of COG8-CDG: A case with novel compound heterozygous mutations diagnosed by targeted exome sequencing.Clin Chim Acta. 2017
223. Whole genome sequencing analyses of Listeria monocytogenes that persisted in a milkshake machine for a year and caused illnesses in Washington State. BMC Microbiol. 2017
224. Whole genome sequencing distinguishes between relapse and reinfection in recurrent leprosy cases. PLoS Negl Trop Dis. 2017

225. Comparative Genomic and Phylogenetic Analysis of a Shiga Toxin Producing Shigella sonnei (STSS) Strain. Front Cell Infect Microbiol. 2017
226. Whole-exome sequencing reveals critical genes underlying metastasis in oesophageal squamous cell carcinoma. J Pathol. 2017
227. Exomic and Epigenomic Analyses in a Pair of Monozygotic Twins Discordant for Cryptorchidism. Twin Res Hum Genet. 2017
228. Pan-cancer analysis reveals technical artifacts in TCGA germline variant calls. BMC Genomics. 2017
229. Loss-of-Function Variants in MYLK Cause Recessive Megacystis Microcolon Intestinal Hypoperistalsis Syndrome. Am J Hum Genet. 2017
230. Investigation of a food-borne outbreak of gastroenteritis in a school canteen revealed a variant of sapovirus genogroup V not detected by standard PCR, Sollentuna, Sweden, 2016. Euro Surveill. 2017
231. Variable expressivity of a likely pathogenic variant in KCNQ2 in a three-generation pedigree presenting with intellectual disability with childhood onset seizures.Am J Med Genet A. 2017
232. The landscape of genetic diseases in Saudi Arabia based on the first 1000 diagnostic panels and exomes. Hum Genet. 2017
233. Search for More Effective Microsatellite Markers for Forensics With Next-Generation Sequencing. IEEE Trans Nanobioscience. 2017
234. Precision oncology based on omics data: The NCT Heidelberg experience. Int J Cancer. 2017
235. Mendel,MD: A user-friendly open-source web tool for analyzing WES and WGS in the diagnosis of patients with Mendelian disorders. PLoS Comput Biol. 2017
236. Validation and Implementation of Clinical Laboratory Improvements Act-Compliant Whole-Genome Sequencing in the Public Health Microbiology Laboratory. J Clin Microbiol. 2017
237. Specific Alleles of CLN7/MFSD8, a Protein That Localizes to Photoreceptor Synaptic Terminals, Cause a Spectrum of Nonsyndromic Retinal Dystrophy. Invest Ophthalmol Vis Sci. 2017
238. Novel EYA1 variants causing Branchio-oto-renal syndrome. Int J Pediatr Otorhinolaryngol. 2017
239. Genetic variation in potential Giardia vaccine candidates cyst wall protein 2 and α1-giardin. Parasitol Res. 2017
240. Molecular epidemiology of Staphylococcus aureus bacteremia in a single large Minnesota medical center in 2015 as assessed using MLST, core genome MLST and spa typing. PLoS One. 2017
241. Detection of clinically relevant copy-number variants by exome sequencing in a large cohort of genetic disorders. Genet Med. 2017
242. The comparison of the performance of four whole genome amplification kits on ion proton platform in copy number variation detection. Biosci Rep. 2017
243. Clinical and molecular characterization of 6 children with glutamate-cysteine ligase deficiency causing hemolytic anemia. Blood Cells Mol Dis. 2017
244. Revealing complete complex KIR haplotypes phased by long-read sequencing technology. Genes Immun. 2017
245. Genetic diagnosis of a Chinese multiple endocrine neoplasia type 2A family through whole genome sequencing. J Biosci. 2017
246. The genetics of gout: towards personalised medicine? BMC Med. 2017
247. Diagnostics and laboratory role in outbreaks. Curr Opin Infect Dis.
248. Clinically Focused Molecular Investigation of 1000 Consecutive Families with Inherited Retinal Disease. Ophthalmology. 2017
249. The Transcriptional Landscape of p53 Signalling Pathway. EBioMedicine. 2017
250. Genome annotation for clinical genomic diagnostics: strengths and weaknesses. Genome Med. 2017
251. Primary Vitreoretinal Lymphoma -- A Review. Asia Pac J Ophthalmol (Phila). 2017
252. Complex and Dynamic Chromosomal Rearrangements in a Family With Seemingly Non-Mendelian Inheritance of Dopa-Responsive Dystonia. JAMA Neurol. 2017
253. Genomic determinants of chronic myelomonocytic leukemia. Leukemia. 2017
254. Genomic diagnosis for children with intellectual disability and/or developmental delay. Genome Med. 2017
255. Whole-Genome Sequencing Coupled to Imputation Discovers Genetic Signals for Anthropometric Traits. Am J Hum Genet. 2017
256. Rapid whole-genome sequencing identifies a novel homozygous NPC1 variant associated with Niemann-Pick type C1 disease in a 7-week-old male with cholestasis. Cold Spring Harb Mol Case Stud. 2017
257. Highly recurrent H3F3A mutations with additional epigenetic regulator alterations in giant cell tumor of bone. Genes Chromosomes Cancer. 2017
258. Matching phenotypes to whole genomes: Lessons learned from four iterations of the personal genome project community challenges. Hum Mutat. 2017

259. Detailed Clinical Phenotype and Molecular Genetic Findings in CLN3-Associated Isolated Retinal Degeneration. JAMA Ophthalmol. 2017
260. Plasmid-free CRISPR/Cas9 genome editing in Plasmodium falciparum confirms mutations conferring resistance to the dihydroisoquinolone clinical candidate SJ733. PLoS One. 2017
261. Whole-genome sequencing of spermatocytic tumors provides insights into the mutational processes operating in the male germline. PLoS One. 2017
262. Emergence of vanA Enterococcus faecium in Denmark, 2005-15. J Antimicrob Chemother. 2017
263. A recessive mutation in beta-IV-spectrin (SPTBN4) associates with congenital myopathy, neuropathy, and central deafness. Hum Genet. 2017
264. Comprehensive whole genome sequence analyses yields novel genetic and structural insights for Intellectual Disability. BMC Genomics. 2017
265. Whole-exome sequencing identifies a novel mutation (R367G) in SCN5A to be associated with familial cardiac conduction disease. Mol Med Rep. 2017
266. Microbiological problems and biofilms associated with Mycobacterium chimaera in heater-cooler units used for cardiopulmonary bypass. J Hosp Infect. 2017
267. Impact of mutations in Toll-like receptor pathway genes on esophageal carcinogenesis. PLoS Genet. 2017
268. Genomic relatedness of Staphylococcus pettenkoferi isolates of different origins. J Med Microbiol. 2017
269. BACH2 immunodeficiency illustrates an association between super-enhancers and haploinsufficiency. Nat Immunol. 2017
270. Sporadic Early-Onset Diffuse Gastric Cancers Have High Frequency of Somatic CDH1 Alterations, but Low Frequency of Somatic RHOA Mutations Compared With Late-Onset Cancers. Gastroenterology. 2017
271. Exome sequencing reveals a de novo POLD1 mutation causing phenotypic variability in mandibular hypoplasia, deafness, progeroid features, and lipodystrophy syndrome (MDPL). Metabolism. 2017
272. Genomic epidemiology of global VIM-producing Enterobacteriaceae. J Antimicrob Chemother. 2017
273. Haploinsufficiency of ZNF462 is associated with craniofacial anomalies, corpus callosum dysgenesis, ptosis, and developmental delay. Eur J Hum Genet. 2017
274. Carriage of extended-spectrum beta-lactamase-producing Enterobacteriaceae in HIV-infected children in Zimbabwe.J Med Microbiol. 2017
275. CAGI4 SickKids clinical genomes challenge: A pipeline for identifying pathogenic variants. Hum Mutat. 2017
276. Population Genomic Analysis of 1,777 Extended-Spectrum Beta-Lactamase-Producing Klebsiella pneumoniae Isolates, Houston, Texas: Unexpected Abundance of Clonal Group 307. MBio. 2017
277. High frequency of silver resistance genes in invasive isolates of Enterobacter and Klebsiella species. J Hosp Infect. 2017
278. Novel OFD1 frameshift mutation in a Chinese boy with Joubert syndrome: a case report and literature review. Clin Dysmorphol. 2017
279. Utility of NIST Whole-Genome Reference Materials for the Technical Validation of a Multigene Next-Generation Sequencing Test. J Mol Diagn. 2017
280. Differential analysis of mutations in the Jewish population and their implications for diseases. Genet Res (Camb). 2017
281. Genome sequence of Shigella flexneri strain SP1, a diarrheal isolate that encodes an extended-spectrum β-lactamase (ESBL). Ann Clin Microbiol Antimicrob. 2017
282. Identification of a missense HOXD13 mutation in a Chinese family with syndactyly type I-c using exome sequencing. Mol Med Rep. 2017
283. Determination of disease phenotypes and pathogenic variants from exome sequence data in the CAGI 4 gene panel challenge. Hum Mutat. 2017
284. Not only dominant, not only optic atrophy: expanding the clinical spectrum associated with OPA1 mutations. Orphanet J Rare Dis. 2017
285. Comprehensive analysis of treatment response phenotypes in rheumatoid arthritis for pharmacogenetic studies. Arthritis Res Ther. 2017
286. Common Variable Immunodeficiency Caused by FANC Mutations. J Clin Immunol. 2017
287. A comprehensive strategy for exome-based preconception carrier screening. Genet Med. 2017
288. Bifidobacterium Bacteremia: Clinical Characteristics and a Genomic Approach To Assess Pathogenicity. J Clin Microbiol. 2017
289. Acquisition and Transmission of Streptococcus pneumoniae Are Facilitated during Rhinovirus Infection in Families with Children. Am J Respir Crit Care Med. 2017
290. Practices and views of neurologists regarding the use of whole-genome sequencing in clinical settings: a web-based survey. Eur J Hum Genet. 2017
291. Whole-genome sequencing identifies homozygous BRCA2 deletion guiding treatment in dedifferentiated prostate cancer. Cold Spring Harb Mol Case Stud. 2017

292. Evolution and dissemination of the Klebsiella pneumoniae clonal group 258 throughout Israeli post-acute care hospitals, 2008-13. J Antimicrob Chemother. 2017
293. Somatic mutation profiles of clear cell endometrial tumors revealed by whole exome and targeted gene sequencing. Cancer. 2017
294. Advances in the role of cytogenetic analysis in the molecular diagnosis of B-cell lymphomas. Expert Rev Mol Diagn. 2017
295. Whole genome characterization of a naturally occurring vancomycin-dependent Enterococcus faecium from a patient with bacteremia. Infect Genet Evol. 2017
296. Complete genome sequencing and clinical analysis of intrahepatic hepatitis B virus cccDNA from HCC. Microb Pathog. 2017
297. Clinical and Molecular Epidemiology of Carbapenem-Resistant Enterobacteriaceae Among Adult Inpatients in Singapore. Clin Infect Dis. 2017
298. MRSA Transmission Dynamics Among Interconnected Acute, Intermediate-Term, and Long-Term Healthcare Facilities in Singapore. Clin Infect Dis. 2017
299. 2015 Epidemic of Severe Streptococcus agalactiae Sequence Type 283 Infections in Singapore Associated With the Consumption of Raw Freshwater Fish: A Detailed Analysis of Clinical, Epidemiological, and Bacterial Sequencing Data. Clin Infect Dis. 2017
300. Genome-wide association study meta-analysis for quantitative ultrasound parameters of bone identifies five novel loci for broadband ultrasound attenuation. Hum Mol Genet. 2017
301. Recessive TAF1A mutations reveal ribosomopathy in siblings with end-stage pediatric dilated cardiomyopathy. Hum Mol Genet. 2017
302. A comparative analysis of whole genome sequencing of esophageal adenocarcinoma pre- and post-chemotherapy. Genome Res. 2017
303. Post-operative atrial fibrillation examined using whole-genome RNA sequencing in human left atrial tissue. BMC Med Genomics. 2017
304. Epidemiology and whole genome sequencing of an ongoing point-source Salmonella Agona outbreak associated with sushi consumption in western Sydney, Australia 2015.Epidemiol Infect. 2017
305. Co-clinical trials demonstrate predictive biomarkers for dovitinib, an FGFR inhibitor, in lung squamous cell carcinoma. Ann Oncol. 2017
306. Hypomorphic mutations in POLR3A are a frequent cause of sporadic and recessive spastic ataxia. Brain. 2017
307. Duplicated Enhancer Region Increases Expression of CTSB and Segregates with Keratolytic Winter Erythema in South African and Norwegian Families. Am J Hum Genet. 2017
308. Streamlining cardiovascular clinical trials to improve efficiency and generalisability. Heart. 2017
309. Lost in translation: returning germline genetic results in genome-scale cancer research. Genome Med. 2017
310. Towards a global cancer knowledge network: dissecting the current international cancer genomic sequencing landscape. Ann Oncol. 2017
311. Epstein-Barr virus-associated gastric cancer reveals intratumoral heterogeneity of PIK3CA mutations. Ann Oncol. 2017
312. Whole Exome Sequencing in Eight Thai Patients With Leber Congenital Amaurosis Reveals Mutations in the CTNNA1 and CYP4V2 Genes. Invest Ophthalmol Vis Sci. 2017
313. Frequent somatic mutations in epigenetic regulators in newly diagnosed chronic myeloid leukemia. Blood Cancer J. 2017
314. A homozygous missense mutation in ERAL1, encoding a mitochondrial rRNA chaperone, causes Perrault syndrome. Hum Mol Genet. 2017
315. A three-caller pipeline for variant analysis of cancer whole-exome sequencing data. Mol Med Rep. 2017
316. CRISPR/Cas9-mediated gene knockout screens and target identification via whole-genome sequencing uncover host genes required for picornavirus infection. J Biol Chem. 2017
317. Prevalence and Outcomes of Achromobacter Species Infections in Adults with Cystic Fibrosis: a North American Cohort Study. J Clin Microbiol. 2017
318. Next-generation Sequencing (NGS) Analysis on Single Circulating Tumor Cells (CTCs) with No Need of Whole-genome Amplification (WGA). Cancer Genomics Proteomics. 2017
319. Investigational drugs for nasopharyngeal carcinoma. Expert Opin Investig Drugs. 2017
320. Tracking the Evolution of Non-Small-Cell Lung Cancer. N Engl J Med. 2017
321. Two novel mutations in ERCC6 cause Cockayne syndrome B in a Chinese family. Mol Med Rep. 2017
322. A novel mutation in pmrB mediates colistin resistance during therapy of Acinetobacter baumannii. Int J Antimicrob Agents. 2017
323. Comprehensive performance comparison of high-resolution array platforms for genome-wide Copy Number Variation (CNV) analysis in humans. BMC Genomics. 2017

324. Whole blood sequencing reveals circulating microRNA associations with high-risk traits in non-ST-segment elevation acute coronary syndrome. Atherosclerosis. 2017
325. The expression of Helicobacter pylori tfs plasticity zone cluster is regulated by pH and adherence, and its composition is associated with differential gastric IL-8 secretion.Helicobacter. 2017
326. Novel compound heterozygous mutations in the PEX1 gene in two Chinese newborns with Zellweger syndrome based on whole exome sequencing. Clin Chim Acta. 2017
327. Whole-genome sequencing suggests mechanisms for 22q11.2 deletion-associated Parkinson's disease. PLoS One. 2017
328. Comprehensive evaluation of genome-wide 5-hydroxymethylcytosine profiling approaches in human DNA. Epigenetics Chromatin. 2017
329. Whole-exome sequencing identifies novel candidate predisposition genes for familial polycythemia vera. Hum Genomics. 2017
330. Clinical implications of genomic profiles in metastatic breast cancer with a focus on TP53 and PIK3CA, the most frequently mutated genes. Oncotarget. 2017
331. The within-host population dynamics of Mycobacterium tuberculosis vary with treatment efficacy. Genome Biol. 2017
332. The importance of the genomic landscape in Waldenström's Macroglobulinemia for targeted therapeutical interventions. Oncotarget. 2017
333. Next generation mapping reveals novel large genomic rearrangements in prostate cancer. Oncotarget. 2017
334. Gene-set analysis shows association between FMRP targets and autism spectrum disorder. Eur J Hum Genet. 2017
335. Integrated genomic analysis of mitochondrial RNA processing in human cancers. Genome Med. 2017
336. Intersect-then-combine approach: improving the performance of somatic variant calling in whole exome sequencing data using multiple aligners and callers. Genome Med. 2017
337. Molecular classification of pulmonary sarcomatoid carcinomas suggests new therapeutic opportunities. Ann Oncol. 2017
338. Cytoplasmic body pathology in severe ACTA1-related myopathy in the absence of typical nemaline rods. Neuromuscul Disord. 2017
339. A phase 2 study of vorinostat in locally advanced, recurrent, or metastatic adenoid cystic carcinoma. Oncotarget. 2017
340. Semantic prioritization of novel causative genomic variants. PLoS Comput Biol. 2017
341. A SLC24A2 Gene Variant Uncovered in Pancreatic Ductal Adenocarcinoma by Whole Exome Sequencing. Tohoku J Exp Med. 2017
342. High prevalence of TP53 mutations is associated with poor survival and an EMT signature in gliosarcoma patients. Exp Mol Med. 2017
343. Tumor immune microenvironment and nivolumab efficacy in EGFR mutation-positive non-small-cell lung cancer based on T790M status after disease progression during EGFR-TKI treatment. Ann Oncol. 2017
344. Non-pncA Gene-Mutated but Pyrazinamide-Resistant Mycobacterium tuberculosis: Why Is That? J Clin Microbiol. 2017
345. Comparison of Whole-Genome Sequencing Methods for Analysis of Three Methicillin-Resistant Staphylococcus aureus Outbreaks. J Clin Microbiol. 2017
346. The genomic potential of the Aspirin in Reducing Events in the Elderly and Statins in Reducing Events in the Elderly studies. Intern Med J. 2017
347. The elusive ideal of inclusiveness: lessons from a worldwide survey of neurologists on the ethical issues raised by whole-genome sequencing. BMC Med Ethics. 2017
348. Ethical considerations surrounding germline next-generation sequencing of children with cancer. Expert Rev Mol Diagn. 2017
349. A rare splice donor mutation in the haptoglobin gene associates with blood lipid levels and coronary artery disease. Hum Mol Genet. 2017
350. A high-throughput molecular data resource for cutaneous neurofibromas. Sci Data. 2017
351. [Cytogenetic and molecular genetic analysis of small supernumerary marker chromosomes in fetal amniotic fluid]. Zhonghua Yi Xue Yi Chuan Xue Za Zhi. 2017
352. Using circulating cell-free DNA to monitor personalized cancer therapy. Crit Rev Clin Lab Sci. 2017
353. Neurogenetic analysis of childhood disintegrative disorder. Mol Autism. 2017
354. A burden of rare variants in BMPR2 and KCNK3 contributes to a risk of familial pulmonary arterial hypertension. BMC Pulm Med. 2017
355. Whole-exome sequencing for prenatal diagnosis of fetuses with congenital anomalies of the kidney and urinary tract.Nephrol Dial Transplant. 2017
356. Monitoring multiple myeloma by quantification of recurrent mutations in serum. Haematologica. 2017
357. Exome Sequence Analysis of 14 Families With High Myopia.Invest Ophthalmol Vis Sci. 2017

358. Candidate Gene Analysis Identifies Mutations in CYP1B1 and LTBP2 in Indian Families with Primary Congenital Glaucoma. Genet Test Mol Biomarkers. 2017
359. Modified Carbapenem Inactivation Method for Phenotypic Detection of Carbapenemase Production among Enterobacteriaceae. J Clin Microbiol. 2017
360. Comprehensive Whole-Genome Sequencing and Reporting of Drug Resistance Profiles on Clinical Cases of Mycobacterium tuberculosis in New York State. J Clin Microbiol. 2017
361. Identification of STXBP2 as a novel susceptibility locus for myocardial infarction in Japanese individuals by an exome-wide association study. Oncotarget. 2017
362. The Mutational Landscape of Circulating Tumor Cells in Multiple Myeloma. Cell Rep. 2017
363. Worldwide Distribution of Cytochrome P450 Alleles: A Meta-analysis of Population-scale Sequencing Projects. Clin Pharmacol Ther. 2017
364. Validation of copy number variation analysis for next-generation sequencing diagnostics. Eur J Hum Genet. 2017
365. The germline variants in DNA repair genes in pediatric medulloblastoma: a challenge for current therapeutic strategies. BMC Cancer. 2017
366. Isolated polycystic liver disease genes define effectors of polycystin-1 function. J Clin Invest. 2017
367. Polycystic Kidney Disease with Hyperinsulinemic Hypoglycemia Caused by a Promoter Mutation in Phosphomannomutase 2. J Am Soc Nephrol. 2017
368. Chlamydia trachomatis ChxR is a transcriptional regulator of virulence factors that function in in vivo host-pathogen interactions. Pathog Dis. 2017
369. Characterization of Human Cytomegalovirus Genome Diversity in Immunocompromised Hosts by Whole-Genome Sequencing Directly From Clinical Specimens. J Infect Dis. 2017
370. Demonstration of Persistent Infections and Genome Stability by Whole-Genome Sequencing of Repeat-Positive, Same-Serovar Chlamydia trachomatis Collected From the Female Genital Tract. J Infect Dis. 2017
371. Genetic Alterations in Esophageal Tissues From Squamous Dysplasia to Carcinoma. Gastroenterology. 2017
372. Advantages of genome sequencing by long-read sequencer using SMRT technology in medical area. Hum Cell. 2017
373. Complex Routes of Nosocomial Vancomycin-Resistant Enterococcus faecium Transmission Revealed by Genome Sequencing. Clin Infect Dis. 2017
374. Development and Validation of Clinical Whole-Exome and Whole-Genome Sequencing for Detection of Germline Variants in Inherited Disease. Arch Pathol Lab Med. 2017
375. Impact of Contaminating DNA in Whole-Genome Amplification Kits Used for Metagenomic Shotgun Sequencing for Infection Diagnosis. J Clin Microbiol. 2017
376. Epidemiological and Molecular Characterization of an Invasive Group A Streptococcus emm32.2 Outbreak. J Clin Microbiol. 2017
377. Screening for familial hypercholesterolaemia in childhood: Avon Longitudinal Study of Parents and Children (ALSPAC). Atherosclerosis. 2017
378. Functional characterization of a common CYP4F11 genetic variant and identification of functionally defective CYP4F11 variants in erythromycin metabolism and 20-HETE synthesis. Arch Biochem Biophys. 2017
379. Global developmental delay and intellectual disability associated with a de novo TOP2B mutation. Clin Chim Acta. 2017
380. The clinical application of NGS-based SNP haplotyping for PGD of Hb H disease. Syst Biol Reprod Med. 2017
381. Clinical characteristics and whole exome/transcriptome sequencing of coexisting chronic myeloid leukemia and myelofibrosis. Am J Hematol. 2017
382. A Zoom-Focus algorithm (ZFA) to locate the optimal testing region for rare variant association tests. Bioinformatics. 2017
383. Genome sequencing and comparative analysis of three hypermucoviscous Klebsiella pneumoniae strains isolated in Russia. Pathog Dis. 2017
384. Intra-individual purifying selection on mitochondrial DNA variants during human oogenesis. Hum Reprod. 2017
385. BCAP31-associated encephalopathy and complex movement disorder mimicking mitochondrial encephalopathy. Am J Med Genet A. 2017
386. Personalized In Vitro and In Vivo Cancer Models to Guide Precision Medicine. Cancer Discov. 2017
387. Defining a Core Genome Multilocus Sequence Typing Scheme for the Global Epidemiology of Vibrio parahaemolyticus. J Clin Microbiol. 2017
388. Outcomes of Diagnostic Exome Sequencing in Patients With Diagnosed or Suspected Autism Spectrum Disorders. Pediatr Neurol. 2017

389. Recessive mutations in SLC35A3 cause early onset epileptic encephalopathy with skeletal defects. Am J Med Genet A. 2017
390. Carbon dating cancer: defining the chronology of metastatic progression in colorectal cancer. Ann Oncol. 2017
391. Insights from early experience of a Rare Disease Genomic Medicine Multidisciplinary Team: a qualitative study.Eur J Hum Genet. 2017
392. Limited role of interferon-kappa (IFNK) truncating mutations in common variable immunodeficiency. Cytokine. 2017
393. Whole-genome sequencing identifies rare genotypes in COMP and CHADL associated with high risk of hip osteoarthritis. Nat Genet. 2017
394. A single-copy Sleeping Beauty transposon mutagenesis screen identifies new PTEN-cooperating tumor suppressor genes.Nat Genet. 2017
395. Mutations in TMEM260 Cause a Pediatric Neurodevelopmental, Cardiac, and Renal Syndrome. Am J Hum Genet. 2017
396. Noninvasive prenatal screening at low fetal fraction: comparing whole-genome sequencing and single-nucleotide polymorphism methods. Prenat Diagn. 2017
397. The genetics of gastroesophageal adenocarcinoma and the use of circulating cell free DNA for disease detection and monitoring.Expert Rev Mol Diagn. 2017
398. Common data elements for clinical research in mitochondrial disease: a National Institute for Neurological Disorders and Stroke project. J Inherit Metab Dis. 2017
399. The Impact of Antibiotic Stewardship Programs in Combating Quinolone Resistance: A Systematic Review and Recommendations for More Efficient Interventions Adv Ther. 2017
400. Real-Time Sequencing of Mycobacterium tuberculosis: Are We There Yet? J Clin Microbiol. 2017
401. Utility of Combining Whole Genome Sequencing with Traditional Investigational Methods To Solve Foodborne Outbreaks of Salmonella Infections Associated with Chicken: A New Tool for Tackling This Challenging Food Vehicle. J Food Prot. 2017
402. [Diagnostics in human genetics : Integration of phenotypic and genomic data]. Bundesgesundheitsblatt Gesundheitsforschung Gesundheitsschutz. 2017
403. Mutations in the Spliceosome Component CWC27 Cause Retinal Degeneration with or without Additional Developmental Anomalies. Am J Hum Genet. 2017
404. Exome sequencing identified a novel SMAD2 mutation in a Chinese family with early onset aortic aneurysms. Clin Chim Acta. 2017
405. Novel and known ribosomal causes of Diamond-Blackfan anaemia identified through comprehensive genomic characterisation. J Med Genet. 2017
406. Characterization of isolates of Eisenbergiella tayi, a strictly anaerobic Gram-stain variable bacillus recovered from human clinical materials in Canada. Anaerobe. 2017
407. Characteristics and changes in invasive meningococcal disease epidemiology in France, 2006-2015. J Infect. 2017
408. Same-Day Diagnostic and Surveillance Data for Tuberculosis via Whole-Genome Sequencing of Direct Respiratory Samples. J Clin Microbiol. 2017
409. Structural Alteration of OmpR as a Source of Ertapenem Resistance in a CTX-M-15-Producing Escherichia coli O25b:H4 Sequence Type 131 Clinical Isolate. Antimicrob Agents Chemother. 2017
410. Whole genome sequencing resource identifies 18 new candidate genes for autism spectrum disorder. Nat Neurosci. 2017
411. Characterisation of invasive clinical Haemophilus influenzae isolates in Queensland, Australia using whole-genome sequencing. Epidemiol Infect. 2017
412. The study of human Y chromosome variation through ancient DNA. Hum Genet. 2017
413. Clinical and molecular phenotyping of a child with Hermansky-Pudlak syndrome-7, an uncommon genetic type of HPS. Mol Genet Metab. 2017
414. Genetics of early-onset Parkinson's disease in Finland: exome sequencing and genome-wide association study. Neurobiol Aging. 2017
415. Homozygous mutations in VAMP1 cause a presynaptic congenital myasthenic syndrome. Ann Neurol. 2017
416. Association of Steroid 5α-Reductase Type 3 Congenital Disorder of Glycosylation With Early-Onset Retinal Dystrophy. JAMA Ophthalmol. 2017.
417. Haploinsufficiency of the E3 ubiquitin-protein ligase gene TRIP12 causes intellectual disability with or without autism spectrum disorders, speech delay, and dysmorphic features.Hum Genet. 2017
418. Haploinsufficiency of the E3 ubiquitin-protein ligase gene TRIP12 causes intellectual disability with or without autism spectrum disorders, speech delay, and dysmorphic features. Jpn J Infect Dis. 2017

419. Characterization of chromosomal abnormalities in pregnancy losses reveals critical genes and loci for human early development. Hum Mutat. 2017
420. Integron-Associated DfrB4, a Previously Uncharacterized Member of the Trimethoprim-Resistant Dihydrofolate Reductase B Family, Is a Clinically Identified Emergent Source of Antibiotic Resistance. Antimicrob Agents Chemother. 2017
421. Two novel mutations in the PPIB gene cause a rare pedigree of osteogenesis imperfecta type IX. Clin Chim Acta. 2017
422. TarSeqQC: Quality control on targeted sequencing experiments in R. Hum Mutat. 2017
423. The role of genomics in common variable immunodeficiency disorders. Clin Exp Immunol. 2017
424. Genomic complexity and targeted genes in anaplastic thyroid cancer cell lines. Endocr Relat Cancer. 2017
425. Homozygous mutation in TXNRD1 is associated with genetic generalized epilepsy. Free Radic Biol Med. 2017
426. Chondrodysplasia with multiple dislocations: comprehensive study of a series of 30 cases. Clin Genet. 2017
427. Short-Read Whole-Genome Sequencing for Laboratory-Based Surveillance of Bordetella pertussis. J Clin Microbiol. 2017
428. Legionellosis acquired through a dental unit: a case study. J Hosp Infect. 2017
429. Analysis of Serial Isolates of mcr-1-Positive Escherichia coli Reveals a Highly Active ISApl1 Transposon. Antimicrob Agents Chemother. 2017
430. Mutations in KIAA0753 cause Joubert syndrome associated with growth hormone deficiency. Hum Genet. 2017
431. Genetic characterization of Polish ccRCC patients: somatic mutation analysis of PBRM1, BAP1 and KDMC5, genomic SNP array analysis in tumor biopsy and preliminary results of chromosome aberrations analysis in plasma cell free DNA. Oncotarget. 2017
432. The glycan-specific sulfotransferase (R77W)GalNAc-4-ST1 putatively responsible for peeling skin syndrome has normal properties consistent with a simple sequence polymorphisim. Glycobiology. 2017
433. Seeding and Establishment of Legionella pneumophila in Hospitals: Implications for Genomic Investigations of Nosocomial Legionnaires' Disease. Clin Infect Dis. 2017
434. Lethal neonatal case and review of primary short-chain enoyl-CoA hydratase (SCEH) deficiency associated with secondary lymphocyte pyruvate dehydrogenase complex (PDC) deficiency. Mol Genet Metab. 2017
435. Genomic profiling of Acute lymphoblastic leukemia in ataxia telangiectasia patients reveals tight link between ATM mutations and chromothripsis. Leukemia. 2017
436. Noninvasive Prenatal Screening of Fetal Aneuploidy without Massively Parallel Sequencing. Clin Chem. 2017
437. Whole genome analysis of porcine astroviruses detected in Japanese pigs reveals genetic diversity and possible intra-genotypic recombination. Infect Genet Evol. 2017
438. Outbreak of KPC-2-producing Enterobacteriaceae caused by clonal dissemination of Klebsiella pneumoniae ST307 carrying an IncX3-type plasmid harboring a truncated Tn4401a. Diagn Microbiol Infect Dis. 2017
439. Validation of risk stratification models in acute myeloid leukemia using sequencing-based molecular profiling. Leukemia. 2017
440. Chromosomally encoded ESBL genes in Escherichia coli of ST38 from Mongolian wild birds. J Antimicrob Chemother. 2017
441. Contribution to Clostridium Difficile Transmission of Symptomatic Patients With Toxigenic Strains Who Are Fecal Toxin Negative. Clin Infect Dis. 2017
442. Clinical and molecular consequences of disease-associated de novo mutations in SATB2. Genet Med. 2017
443. Clonal or not clonal? Investigating hospital outbreaks of KPC-producing Klebsiella pneumoniae with whole-genome sequencing. Clin Microbiol Infect. 2017
444. Nosocomial transmission of carbapenem-resistant Klebsiella pneumoniae elucidated by single-nucleotide variation analysis: a case investigation. Infection. 2017
445. A Whole-Genome Sequencing Approach To Study Cefoxitin-Resistant Salmonella enterica Serovar Heidelberg Isolates from Various Sources. Antimicrob Agents Chemother. 2017
446. Recent advances in biochemical and molecular diagnostics for the rapid detection of antibiotic-resistant Enterobacteriaceae: a focus on ß-lactam resistance. Expert Rev Mol Diagn. 2017
447. Genome-Wide Analysis Identifies MEN1 and MAX Mutations and a Neuroendocrine-Like Molecular Heterogeneity in Quadruple WT GIST. Mol Cancer Res. 2017
448. GUCA1A mutation causes maculopathy in a five-generation family with a wide spectrum of severity. Genet Med. 2017
449. Molecular genetic findings and clinical correlations in 100 patients with Joubert syndrome and related disorders prospectively evaluated at a single center. Genet Med. 2017

450. Prospective comparison of the cost-effectiveness of clinical whole-exome sequencing with that of usual care overwhelmingly supports early use and reimbursement. Genet Med. 2017
451. Frequent inactivating germline mutations in DNA repair genes in patients with Ewing sarcoma. Genet Med. 2017
452. Environmental emission of multiresistant Escherichia coli carrying the colistin resistance gene mcr-1 from German swine farms. J Antimicrob Chemother. 2017.
453. STAG1 mutations cause a novel cohesinopathy characterised by unspecific syndromic intellectual disability. J Med Genet. 2017
454. Robust and rapid algorithms facilitate large-scale whole genome sequencing downstream analysis in an integrative framework. Nucleic Acids Res. 2017
455. Characterization of Class IIa Bacteriocin Resistance in Enterococcus faecium. Antimicrob Agents Chemother. 2017
456. Myb-like, SWIRM, and MPN domains 1 (MYSM1) deficiency: Genotoxic stress-associated bone marrow failure and developmental aberrations. J Allergy Clin Immunol. 2017
457. Two novel candidate genes identified in adults from the Newfoundland population with addictive tendencies towards food. Appetite. 2017
458. Outcomes, infectiousness, and transmission dynamics of patients with extensively drug-resistant tuberculosis and home-discharged patients with programmatically incurable tuberculosis: a prospective cohort study.Lancet Respir Med. 2017
459. Fatal sepsis caused by multidrug-resistant Bacteroides fragilis, harboring a cfiA gene and an upstream insertion sequence element, in Japan. Anaerobe. 2017
460. Haemagglutinin and neuraminidase sequencing delineate nosocomial influenza outbreaks with accuracy equivalent to whole genome sequencing. J Infect. 2017
461. A taxonomy of medical uncertainties in clinical genome sequencing. Genet Med. 2017
462. Clinical utility gene card for: Non-Syndromic Microphthalmia Including Next-Generation Sequencing-Based Approaches. Eur J Hum Genet. 2017
463. Dissemination of blaOXA-58 in Proteus mirabilis isolates from Germany. J Antimicrob Chemother. 2017
464. Application of whole exome sequencing in elucidating the phenotype and genotype spectrum of junctional epidermolysis bullosa: A preliminary experience of a tertiary care centre in India. J Dermatol Sci. 2017
465. A Large Inversion Involving GNAS Exon A/B and All Exons Encoding Gsα Is Associated With Autosomal Dominant Pseudohypoparathyroidism Type Ib (PHP1B). J Bone Miner Res. 2017
466. Norwegian patients and retail chicken meat share cephalosporin-resistant Escherichia coli and IncK/blaCMY-2 resistance plasmids. Clin Microbiol Infect. 2017
467. Characterization of carbapenemase-producing Enterobacteriaceae in the West Midlands region of England: 2007-14. J Antimicrob Chemother. 2017
468. Pancreatic cancer cell lines as patient-derived avatars: genetic characterisation and functional utility. Gut. 2018
469. The primacy of NF1 loss as the driver of tumorigenesis in neurofibromatosis type 1-associated plexiform neurofibromas. Oncogene. 2017
470. Translating genomic profiling to gastrointestinal cancer treatment. Future Oncol. 2017
471. Genetic and biochemical characterization of HMB-1, a novel subclass B1 metallo-β-lactamase found in a Pseudomonas aeruginosa clinical isolate. J Antimicrob Chemother. 2017
472. Identification and functional characterization of a novel MTFMT mutation associated with selective vulnerability of the visual pathway and a mild neurological phenotype. Neurogenetics. 2017
473. FEELnc: a tool for long non-coding RNA annotation and its application to the dog transcriptome. Nucleic Acids Res. 2017
474. Whole-Genome Bisulfite Sequencing of Human Pancreatic Islets Reveals Novel Differentially Methylated Regions in Type 2 Diabetes Pathogenesis. Diabetes. 2017
475. Discordance of Somatic Mutations Between Asian and Caucasian Patient Populations with Gastric Cancer. Mol Diagn Ther. 2017
476. Evaluation of the Myocilin Mutation Gln368Stop Demonstrates Reduced Penetrance for Glaucoma in European Populations. Ophthalmology. 2017
477. Whole genome sequencing reveals a 7 base-pair deletion in DMD exon 42 in a dog with muscular dystrophy. Mamm Genome. 2017
478. Genetic heterogeneity in Pakistani microcephaly families revisited. Clin Genet. 2017
479. Emergence and dissemination of a linezolid-resistant Staphylococcus capitis clone in Europe. J Antimicrob Chemother. 2017
480. Benzalkonium tolerance genes and outcome in Listeria monocytogenes meningitis. Clin Microbiol Infect. 2017

481. Morphological characterization of colorectal cancers in The Cancer Genome Atlas reveals distinct morphology-molecular associations: clinical and biological implications. Mod Pathol. 2017
482. 17q21.31 duplication causes prominent tau-related dementia with increased MAPT expression. Mol Psychiatry. 2017
483. Abnormal hematopoiesis and autoimmunity in human subjects with germline IKZF1 mutations. J Allergy Clin Immunol. 2017
484. Global DNA methylation profiling of manganese-exposed human neuroblastoma SH-SY5Y cells reveals epigenetic alterations in Parkinson's disease-associated genes. Arch Toxicol. 2017
485. Genomic analysis reveals the presence of a class D beta-lactamase with broad substrate specificity in animal bite associated Capnocytophaga species. Eur J Clin Microbiol Infect Dis. 2017
486. A lethal neonatal phenotype of mitochondrial short-chain enoyl-CoA hydratase-1 deficiency. Clin Genet. 2017
487. Identification of novel genes by whole-exome sequencing can improve gastric cancer precision oncology. Future Oncol. 2017
488. Vitamin A deficiency due to bi-allelic mutation of RBP4: There's more to it than meets the eye. Ophthalmic Genet. 2017
489. Bi-allelic IARS mutations in a child with intra-uterine growth retardation, neonatal cholestasis, and mild developmental delay. Clin Genet. 2017
490. Diagnostic value of exome and whole genome sequencing in craniosynostosis. J Med Genet. 2017
491. Diagnostic challenge of Diamond-Blackfan anemia in mothers and children by whole-exome sequencing. Int J Hematol. 2017
492. My Identical Twin Sequenced our Genome. J Genet Couns. 2017
493. Genomic Evolution after Chemoradiotherapy in Anal Squamous Cell Carcinoma. Clin Cancer Res. 2017
494. Identification of a rare germline NBN gene mutation by whole exome sequencing in a lung-cancer survivor from a large family with various types of cancer. Fam Cancer. 2017
495. Genomic Heterogeneity and Exceptional Response to Dual Pathway Inhibition in Anaplastic Thyroid Cancer. Clin Cancer Res. 2017
496. The cancer precision medicine knowledge base for structured clinical-grade mutations and interpretations. J Am Med Inform Assoc. 2017
497. Association of Distinct Mutational Signatures With Correlates of Increased Immune Activity in Pancreatic Ductal Adenocarcinoma. JAMA Oncol. 2017
498. Renal cell carcinoma harboring somatic TSC2 mutations in a child with methylmalonic acidemia. Pediatr Blood Cancer. 2017
499. HSV-1 clinical isolates with unique in vivo and in vitro phenotypes and insight into genomic differences. J Neurovirol. 2017
500. Primary familial brain calcification linked to deletion of 5' noncoding region of SLC20A2. Acta Neurol Scand. 2017
501. Loss of the arginine methyltranserase PRMT7 causes syndromic intellectual disability with microcephaly and brachydactyly. Clin Genet. 2017
502. Numerous Brugada syndrome-associated genetic variants have no effect on J-point elevation, syncope susceptibility, malignant cardiac arrhythmia, and all-cause mortality. Genet Med. 2017
503. Increasing the sensitivity of clinical exome sequencing through improved filtration strategy. Genet Med. 2017
504. Landscape of Genomic Alterations in Pituitary Adenomas. Clin Cancer Res. 2017
505. Whole-exome sequencing in the molecular diagnosis of individuals with congenital anomalies of the kidney and urinary tract and identification of a new causative gene. Genet Med. 2017
506. Molecular Differences between Screen-Detected and Interval Breast Cancers Are Largely Explained by PAM50 Subtypes. Clin Cancer Res. 2017
507. Clinical utility of non-invasive prenatal testing in pregnancies with ultrasound anomalies. Ultrasound Obstet Gynecol. 2017
508. Clinical application of whole-genome low-coverage next-generation sequencing to detect and characterize balanced chromosomal translocations. Clin Genet. 2017
509. Exome sequencing of Pakistani consanguineous families identifies 30 novel candidate genes for recessive intellectual disability. Mol Psychiatry. 2017
510. A balanced translocation disrupting BCL2L10 and PNLDC1 segregates with affective psychosis. Am J Med Genet B Neuropsychiatr Genet. 2017
511. The use of whole exome sequencing for the diagnosis of autosomal recessive malignant infantile osteopetrosis. Clin Genet. 2017
512. Gene Expression Profiling of Tumors From Heavily Pretreated Patients With Metastatic Cancer for the Selection of Therapy: A Pilot Study. Am J Clin Oncol. 20117

Supplementary Information 2 – Summary of literature search

Publication number	Subject of publication
1	infectious diseases
2	review
3	clinically relevant genetic variants
4	WES
5	WES
6	infectious diseases
7	review
8	review
9	cancer
10	cancer
11	cancer
12	cancer
13	WES
14	WES
15	WES
16	WES
17	clinically relevant genetic variants
18	infectious diseases
19	WES
20	clinically relevant genetic variants
21	infectious diseases
22	N/A
23	WES
24	infectious diseases
25	infectious diseases
26	review
27	WES
28	pharmacogenetics
29	WES
30	N/A
31	WES
32	clinically relevant

Publication number	Subject of publication
	genetic variants
33	N/A
34	infectious diseases
35	WES
36	clinically relevant genetic variants
37	clinically relevant genetic variants
38	infectious diseases
39	N/A
40	infectious diseases
41	clinically relevant genetic variants
42	WES
43	cancer
44	infectious diseases
45	WES
46	cancer
47	cancer
48	WES
49	review
50	infectious diseases
51	review
52	WES
53	WES
54	clinically relevant genetic variants
55	clinically relevant genetic variants
56	newborn testing
57	infectious diseases
58	WES
59	WES
60	infectious diseases
61	infectious diseases
62	N/A

Publication number	Subject of publication
63	WES
64	cancer
65	infectious diseases
66	N/A
67	WES
68	cancer
69	WES
70	WES
71	WES
72	WES
73	cancer
74	clinically relevant genetic variants
75	infectious diseases
76	WES
77	N/A
78	clinically relevant genetic variants
79	N/A
80	infectious diseases
81	N/A
82	WES
83	infectious diseases
84	N/A
85	review
86	WES
87	N/A
88	infectious diseases
89	newborn testing
90	WES
91	population study
92	infectious diseases
93	WES
94	N/A
95	infectious diseases
96	N/A

Publication number	Subject of publication
97	review
98	N/A
99	clinically relevant genetic variants
100	cancer
101	review
102	infectious diseases
103	review
104	infectious diseases
105	WES
106	infectious diseases
107	N/A
108	N/A
109	N/A
110	infectious diseases
111	WES
112	N/A
113	N/A
114	review
115	WES
116	clinically relevant genetic variants
117	N/A
118	N/A
119	N/A
120	infectious diseases
121	clinically relevant genetic variants
122	review
123	WES
124	clinically relevant genetic variants
125	N/A
126	review
127	N/A
128	infectious diseases
129	N/A

Publication number	Subject of publication
130	N/A
131	N/A
132	review
133	N/A
134	N/A
135	N/A
136	review
137	N/A
138	N/A
139	WES
140	N/A
141	N/A
142	N/A
143	N/A
144	infectious diseases
145	WES
146	N/A
147	WES
148	infectious diseases
149	clinically relevant genetic variants
150	WES
151	infectious diseases
152	WES
153	N/A
154	N/A
155	infectious diseases
156	WES
157	population study
158	N/A
159	N/A
160	WES
161	WES
162	N/A
163	N/A
164	N/A
165	infectious diseases
166	cancer

Publication number	Subject of publication
167	N/A
168	N/A
169	review
170	review
171	clinically relevant genetic variants
172	cancer
173	N/A
174	infectious diseases
175	WES
176	N/A
177	infectious diseases
178	N/A
179	cancer
180	WES
181	review
182	WES
183	infectious diseases
184	WES
185	N/A
186	N/A
187	clinically relevant genetic variants
188	WES
189	cancer
190	N/A
191	WES
192	infectious diseases
193	N/A
194	N/A
195	infectious diseases
196	N/A
197	N/A
198	review
199	WES
200	infectious diseases
201	infectious

Publication number	Subject of publication
	diseases
202	clinically relevant genetic variants
203	infectious diseases
204	N/A
205	WES
206	review
207	cancer
208	N/A
209	N/A
210	infectious diseases
211	infectious diseases
212	cancer
213	WES
214	N/A
215	review
216	N/A
217	infectious diseases
218	infectious diseases
219	N/A
220	infectious diseases
221	infectious diseases
222	N/A
223	infectious diseases
224	clinically relevant genetic variants
225	infectious diseases
226	WES
227	N/A
228	N/A
229	WES
230	infectious diseases
231	WES
232	WES

Publication number	Subject of publication
233	forensic medicine
234	N/A
235	N/A
236	N/A
237	clinically relevant genetic variants
238	WES
239	infectious diseases
240	infectious diseases
241	WES
242	N/A
243	clinically relevant genetic variants
244	N/A
245	clinically relevant genetic variants
246	review
247	review
248	clinically relevant genetic variants
249	N/A
250	review
251	review
252	clinically relevant genetic variants
253	WES
254	clinically relevant genetic variants
255	clinically relevant genetic variants
256	N/A
257	WES
258	review
259	clinically relevant genetic variants
260	CRISPR
261	cancer
262	infectious diseases
263	N/A
264	clinically relevant genetic variants

Publication number	Subject of publication
265	WES
266	infectious diseases
267	cancer
268	infectious diseases
269	clinically relevant genetic variants
270	WES
271	WES
272	infectious diseases
273	WES
274	infectious diseases
275	N/A
276	infectious diseases
277	infectious diseases
278	N/A
279	N/A
280	WES
281	infectious diseases
282	WES
283	WES
284	N/A
285	clinically relevant genetic variants
286	WES
287	WES
288	infectious diseases
289	infectious diseases
290	N/A
291	cancer
292	infectious diseases
293	WES
294	review
295	infectious diseases

Publication number	Subject of publication
296	infectious diseases
297	infectious diseases
298	infectious diseases
299	infectious diseases
300	clinically relevant genetic variants
301	WES
302	WES
303	N/A
304	infectious diseases
305	WES
306	WES
307	clinically relevant genetic variants
308	review
309	cancer
310	N/A
311	N/A
312	WES
313	cancer
314	WES
315	N/A
316	CRISPR (basic research)
317	infectious diseases
318	N/A
319	cancer
320	cancer
321	N/A
322	infectious diseases
323	N/A
324	N/A
325	infectious diseases
326	WES
327	clinically relevant genetic variants

Publication number	Subject of publication
328	N/A
329	WES
330	WES
331	infectious diseases
332	WES
333	cancer
334	N/A
335	N/A
336	N/A
337	WES
338	N/A
339	WES
340	N/A
341	WES
342	WES
343	WES
344	infectious diseases
345	N/A
346	WES
347	N/A
348	N/A
349	clinically relevant genetic variants
350	cancer
351	prenatal screening
352	cancer
353	WES
354	clinically relevant genetic variants
355	WES
356	N/A
357	WES
358	WES
359	infectious diseases
360	infectious diseases
361	WES
362	cancer
363	population study

Publication number	Subject of publication
364	N/A
365	N/A
366	WES
367	clinically relevant genetic variants
368	infectious diseases
369	infectious diseases
370	infectious diseases
371	cancer
372	review
373	infectious diseases
374	clinically relevant genetic variants
375	N/A
376	infectious diseases
377	clinically relevant genetic variants
378	WES
379	N/A
380	N/A
381	WES
382	N/A
383	infectious diseases
384	N/A
385	N/A
386	review
387	infectious diseases
388	WES
389	N/A
390	cancer
391	review
392	N/A
393	clinically relevant genetic variants
394	N/A
395	WES
396	prenatal

Publication number	Subject of publication
	screening
397	review
398	N/A
399	review
400	review
401	infectious diseases
402	N/A
403	WES
404	WES
405	clinically relevant genetic variants
406	infectious diseases
407	infectious diseases
408	infectious diseases
409	infectious diseases
410	clinically relevant genetic variants
411	infectious diseases
412	review
413	N/A
414	clinically relevant genetic variants
415	clinically relevant genetic variants
416	clinically relevant genetic variants
417	WES
418	infectious diseases
419	preimplantation genetic testing
420	infectious diseases
421	WES
422	N/A
423	review
424	WES
425	WES

Publication number	Subject of publication
426	WES
427	infectious diseases
428	N/A
429	infectious diseases
430	WES
431	cancer
432	WES
433	infectious diseases
434	WES
435	cancer
436	N/A
437	N/A
438	infectious diseases
439	N/A
440	infectious diseases
441	infectious diseases
442	N/A
443	infectious diseases
444	infectious diseases
445	infectious diseases
446	infectious diseases
447	WES
448	N/A
449	N/A
450	WES
451	cancer
452	infectious diseases
453	clinically relevant genetic variants
454	N/A
455	infectious diseases
456	N/A

Publication number	Subject of publication
457	WES
458	infectious diseases
459	infectious diseases
460	infectious diseases
461	infectious diseases
462	N/A
463	infectious diseases
464	WES
465	N/A
466	infectious diseases
467	infectious diseases
468	WES
469	N/A
470	review
471	infectious diseases
472	N/A
473	N/A
474	N/A
475	N/A
476	N/A
477	N/A
478	WES
479	infectious diseases
480	infectious diseases
481	WES
482	N/A
483	WES
484	N/A
485	infectious diseases
486	N/A
487	WES
488	N/A
489	N/A

Publication number	Subject of publication
490	clinically relevant genetic variants
491	WES
492	N/A
493	WES
494	WES
495	WES
496	N/A
497	cancer
498	N/A
499	infectious diseases
500	clinically relevant genetic variants
501	N/A
502	WES
503	N/A
504	WES
505	WES
506	cancer
507	prenatal screening
508	clinically relevant genetic variants
509	WES
510	clinically relevant genetic variants
511	WES
512	N/A

Supplementary Information 3 – WGS timeline

year	number of records
2017	1890
2016	1707
2015	1532
2014	1232
2013	852
2012	644
2011	349
2010	231
2009	164
2008	88
2007	86

Supplementary Information 4 – False positive and false negative variants - coverage

Depth statistics for false negative variant calls (30×)

Caller	1st quartile	Median	Mean	3rd quartile
Deep Variant 0.4.1	14.00	19.00	19.20	23.00
SpeedSeq 0.1.0	11.00	14.00	14.46	18.00
GATK 4.0	5.00	7.00	7.85	10.00

Depth statistics for false positive variant calls (30×)

Caller	1st quartile	Median	Mean	3rd quartile
Deep Variant 0.4.1	16.00	21.00	21.14	25.00
SpeedSeq 0.1.0	13.00	18.00	21.82	24.00
GATK 4.0	9.00	15.00	17.10	23.00

Supplementary Information 5 – False positive and false negative variants - context

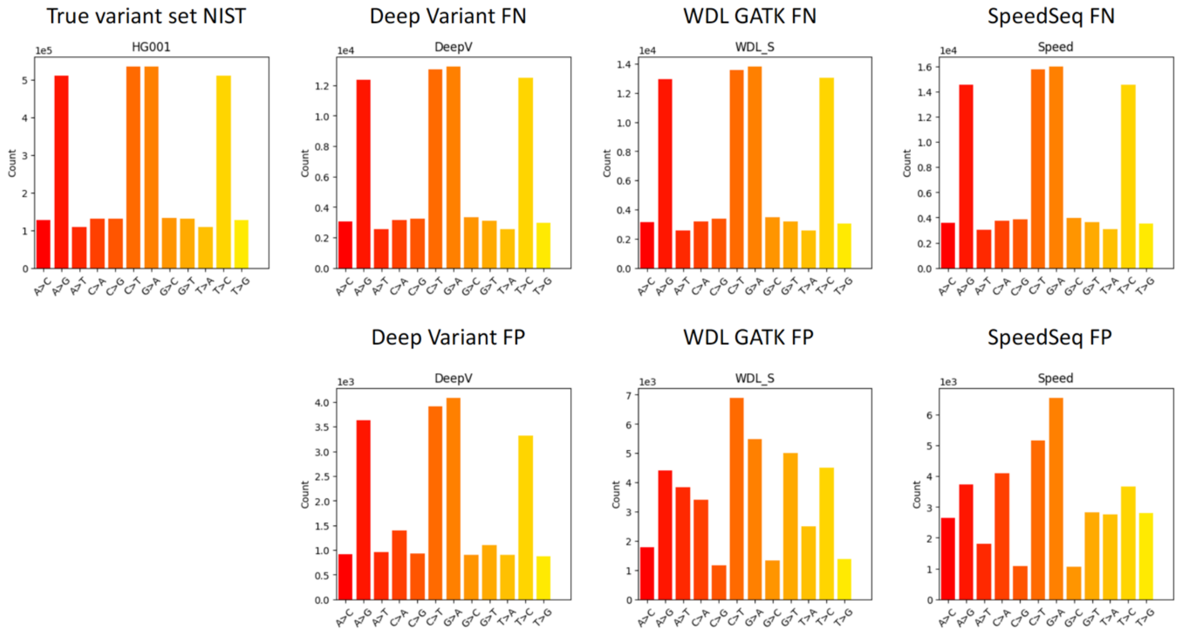

Supplement: Supplementary file 1 — Supplementary Information [file 41598_2018_36177_MOESM1_ESM.pdf]
